# Characterizing Performance on a Suite of English-Language NeuroUX Mobile Cognitive Tests in a US Adult Sample: Ecological Momentary Cognitive Testing Study

**DOI:** 10.2196/51978

**Published:** 2024-11-25

**Authors:** Emily W Paolillo, Jessica Bomyea, Colin A Depp, Ashley M Henneghan, Anunay Raj, Raeanne C Moore

**Affiliations:** 1 Department of Neurology, Weill Institute for Neurosciences, University of California, San Francisco San Francisco, CA United States; 2 Department of Psychiatry, University of California San Diego School of Medicine La Jolla, CA United States; 3 VA San Diego Healthcare System La Jolla, CA United States; 4 School of Nursing University of Texas at Austin Austin, TX United States; 5 NeuroUX, Inc. Dover, DE United States

**Keywords:** digital health, cognition, cognitive aging, neuropsychology, mobile health, psychometrics, mobile phone, Ecological Momentary Assessment, EMA, NeuroUX

## Abstract

**Background:**

Mobile cognitive testing is growing in popularity, with numerous advantages over traditional cognitive testing; however, the field lacks studies that deeply examine mobile cognitive test data from general adult samples.

**Objective:**

This study characterized performance for a suite of 8 mobile cognitive tests from the NeuroUX platform in a sample of US adults across the adult lifespan.

**Methods:**

Overall, 393 participants completed 8 NeuroUX cognitive tests and a brief ecological momentary assessment survey once per day on their smartphones for 10 consecutive days; each test was administered 5 times over the testing period. The tests tapped the domains of executive function, processing speed, reaction time, recognition memory, and working memory. Participants also completed a poststudy usability feedback survey. We examined alternate form test-retest reliability; practice effects; and associations between scores (averages and intraindividual variability) and demographics as well as test-taking context (ie, smartphone type, being at home vs not at home, and being alone vs not alone).

**Results:**

Our final sample consisted of 393 English-speaking US residents (aged 20-79 y; female: n=198, 50.4%). Of the 367 participants who provided responses about their race and ethnicity, 258 (70.3%) were White. Of the 393 participants, 181 (46.1%) were iOS users, and 212 (53.9%) were Android users. Of 12 test scores derived from the 8 tests, 9 (75%) showed good to excellent test-retest reliability (intraclass correlation coefficients >0.76). Practice effects (ie, improvements in performance) were observed for 4 (33%) of the 12 scores. Older age was associated with worse performance on most of the test scores (9/12, 75%) and greater within-person variability for nearly all reaction time scores (3/4, 75%). Relationships with smartphone type showed better performance among iOS users and those with newer Android software versions compared to those with older software. Being at home (vs not at home) was associated with better performance on tests of processing speed. Being alone (vs not alone) was associated with better performance on tests of recognition and working memory. Poststudy feedback indicated that participants found NeuroUX easy to learn and use, an enjoyable experience, and an app that would be helpful in understanding their thinking skills. Only 4.2% (16/379) endorsed privacy concerns, and 77.3% (293/379) reported that they would be willing to share their results with their health care provider. Older age—but not other demographics—was associated with finding the tests more challenging.

**Conclusions:**

In a sample of adults across a wide age range, this study characterized features that are particularly important for the interpretation of remote, repeated mobile cognitive testing performance, including test-retest reliability, practice effects, smartphone type, and test-taking context. These data enhance the understanding and application of mobile cognitive testing, paving the way for improved clinical decision-making, personalized interventions, and advancements in cognitive research.

## Introduction

### Background

Advances in smartphone technology have allowed researchers to couple brief cognitive testing with frequently repeated ecological momentary assessment (EMA), opening the door to studying cognitive performance as it occurs in the natural environment [1]. Mobile cognitive testing in the context of an EMA paradigm (also known as ecological momentary cognitive testing) has several advantages that enhance cognitive research, including the ability to (1) collect data as people go about their daily lives, (2) randomly sample cognitive assessments across various times and occasions to examine an individual’s average performance and variability in performance, and (3) measure cognition alongside mood and behavior in real time and across the different contexts of daily life [2].

Traditionally, clinicians and researchers have relied on standardized paper-and-pencil tests or laboratory-based experiments to measure cognitive abilities. While these methods are gold standard assessments of cognitive functioning, they also have some limitations; for example, laboratory or clinical-based tests can be resource intensive because evaluations typically last >1 hour and require trained clinicians to administer and interpret the testing results [3]. They are also conducted in controlled (ie, artificial) testing environments, which may lack ecological validity [4]. Mobile cognitive tests are increasingly being used to assess cognitive performance as a method to enhance measurement of real-world cognitive function either in conjunction with traditional neuropsychological testing or as a stand-alone assessment [5]. This remote assessment method offers unique advantages over traditional neuropsychological testing in specific cases, including studying scientific questions related to cognitive performance in a naturalistic environment or acute fluctuations in cognitive performance [6]. It can also increase access to neuropsychological care by reducing geographic or mobility barriers for individuals [7]. Growing research has also shown that mobile cognitive testing is reliable and may be more sensitive to subtle or early cognitive deficits than single-administration neuropsychological tests [1,8-10].

Despite the growing popularity of mobile cognitive tests, there are challenges in the validation of these tests. For one, there is a notable lack of comprehensive data available from general, nonclinical populations for these assessment tools. These data provide a frame of reference for interpreting individual performance and enable comparisons across clinical populations when demographics are appropriately matched. They serve as a benchmark against which an individual’s cognitive abilities may be evaluated, aiding in diagnostic decision-making, personalized interventions, and the identification of cognitive strengths and weaknesses. In a 2017 systematic review of the applications of mobile cognitive testing in clinical research, 12 articles were identified that used, and reported the psychometric properties of, self-administered mobile cognitive tests [1]. Since then, other groups have published additional work on the feasibility, reliability, and validity of different mobile cognitive testing platforms in small to midsize samples of various populations, including healthy adults [9,11]; healthy older adults [12]; and older adults at risk for Alzheimer disease (AD) [13], frontotemporal dementia [7], and Parkinson disease [14,15]. Second, as mobile cognitive tests produce within-person data, additional psychometric properties concerning within-person variability are possible that are not possible in single-administration cognitive tests; for example, Aschenbrenner et al [16] examined within-person variability in test performance across 28 repeated sessions of a mobile cognitive testing platform, administered over a 1-week period, and found increased variability on a processing speed task among persons at risk for AD compared to those without AD risk. In addition, with mobile cognitive tests, practice effects are measured over a far briefer period than in traditional tests, and a better understanding of these practice effect trends in healthy samples is needed. Third and last, because mobile cognitive tests are self-administered as participants go about their daily lives, context, such as the influence of being alone versus not alone and being at home versus not at home, needs to be considered when examining test performance.

### Objectives

In our prior work in recent years, we have published on the psychometric properties of several of the NeuroUX mobile cognitive tests in clinical populations, including people with schizophrenia [17,18], those with bipolar disorder [17-19], and individuals with mild cognitive impairment [20]. The primary objective of this paper was to present data for a mobile cognitive testing protocol that includes a suite of 8 NeuroUX mobile cognitive tests, derived from a relatively large sample (n=393) of adults in the United States ranging in age from 20 to 79 years. Participants completed EMA surveys and mobile cognitive testing once daily for 10 days. The cognitive domains assessed included tests of processing speed, working memory, recognition memory, executive function, and reaction time. We present the distributions of scores for each test (averaged across study days), along with data on alternate form test-retest reliability, practice effects, and associations between scores (both averages and intraindividual variability) and demographics as well as test-taking context (ie, smartphone type, being at home vs not at home, being alone vs not alone, and the time of day). In addition, we report scores for each test by age bin and present usability feedback data from the participants.

## Methods

### Participants

A total of 394 participants from the United States were recruited via an opt-in, convenience sampling approach using Prolific, a web-based research and data collection platform (Prolific Academic Ltd). Prolific provides researchers access to a pool of >130,000 participants whose identities are verified using email, telephone number, and proof of identity (eg, driver’s license), allowing quick and efficient remote data collection. Participants were recruited to Prolific through word of mouth—such as referrals from other Prolific participants—as well as through social media shares and flyers distributed on university campuses. Participants then sign up on Prolific through their web-based portal and enroll in various studies per individual preference. Each study must communicate the following to interested participants: the purpose of the research and the intended use of the collected data. Participants are fully anonymized, and Prolific provides an anonymized internal messaging service, which allows participants to message researchers (and vice versa). Researchers cannot access participants’ identifiable information, and Prolific does not store any data provided within studies.

The inclusion criteria for this study included being aged between 20 and 79 years, having no diagnosis of cognitive impairment (per self-report), being of US nationality, being born in the United States, currently residing in the United States, and having English as the first language. No exclusionary criteria were applied. The target recruitment goal was 300 participants, with 50 participants per age decade (20-29 y, 30-39 y, etc), which was based on estimates from pervious similar short-term, repeated cognitive assessment studies. After careful review of the data, of the 394 participants recruited, 1 (0.3%) was excluded due to performing at the floor level of all NeuroUX tests, leaving 393 (99.7%) adults in the final sample.

### Procedures

Participants completed 4 of the 8 NeuroUX cognitive tests and a brief EMA survey once per day on their personal smartphones for 10 consecutive days. Each daily session took 8 to 10 minutes to complete, and the link to complete the daily session was active from 7 AM to 8 PM, allowing participants flexibility to complete the tasks at their convenience over the course of the day. Each of the 8 NeuroUX cognitive tests described in this study was completed 5 times (Table 1). There are multiple versions of each test, and alternate forms of each test were used for each of the 5 administrations. To inform the context in which participants were completing the cognitive tests, daily EMA surveys asked participants to report their current location (being at home vs not at home) and whether they were alone or with others, as well as rate their mood. At the end of each session, participants completed EMA questions asking whether they were interrupted or distracted during the testing session. At the conclusion of the 10 days of testing, participants were sent a poststudy feedback survey, which asked questions about their experience with NeuroUX (answered with Likert-scale response options ranging from *strongly agree* to *strongly disagree*), whether they had privacy concerns related to their NeuroUX use, and whether they would be willing to share NeuroUX data with their health care provider.

**Table 1:** NeuroUX test administration protocol for this study in US adults.

Cognitive tests	Study day									
	1	2	3	4	5	6	7	8	9	10
Memory List	—^a^	2nd^b^	1st	—	1st	—	—	2nd	2nd	—
Memory Matrix	2nd	—	4th	—	—	3rd	1st	—	4th	—
Matching Pair	1st	—	—	2nd	—	2nd	—	4th	1st	—
Quick Tap 1	—	3rd	—	3rd	2nd	—	—	1st	—	2nd
Quick Tap 2	—	4th	—	4th	3rd	—	—	3rd	—	3rd
Odd One Out	3rd	—	2nd	—	4th	—	4th	—	—	1st
CopyKat	4th	—	3rd	—	—	1st	2nd	—	3rd	—
Hand Swype	—	1st	—	1st	—	4th	3rd	—	—	4th

^a^Not applicable.

^b^Values within the table represent the order in which the tests were administered on each study day. On the days when Memory List was administered, the recall portion was always administered at the end of the testing session.

### Measures

#### Overview

NeuroUX is a proprietary platform designed to deliver mobile cognitive tests and EMA surveys. This platform has been implemented in several patient populations, including individuals with serious mental illness, older adults with mild cognitive impairment, and persons with cancer-related cognitive impairment [17,19-21]. Eight cognitive tests, as well as a poststudy feedback survey, described in the following subsections, were administered as part of this study.

#### Memory List

**Figure 1:**
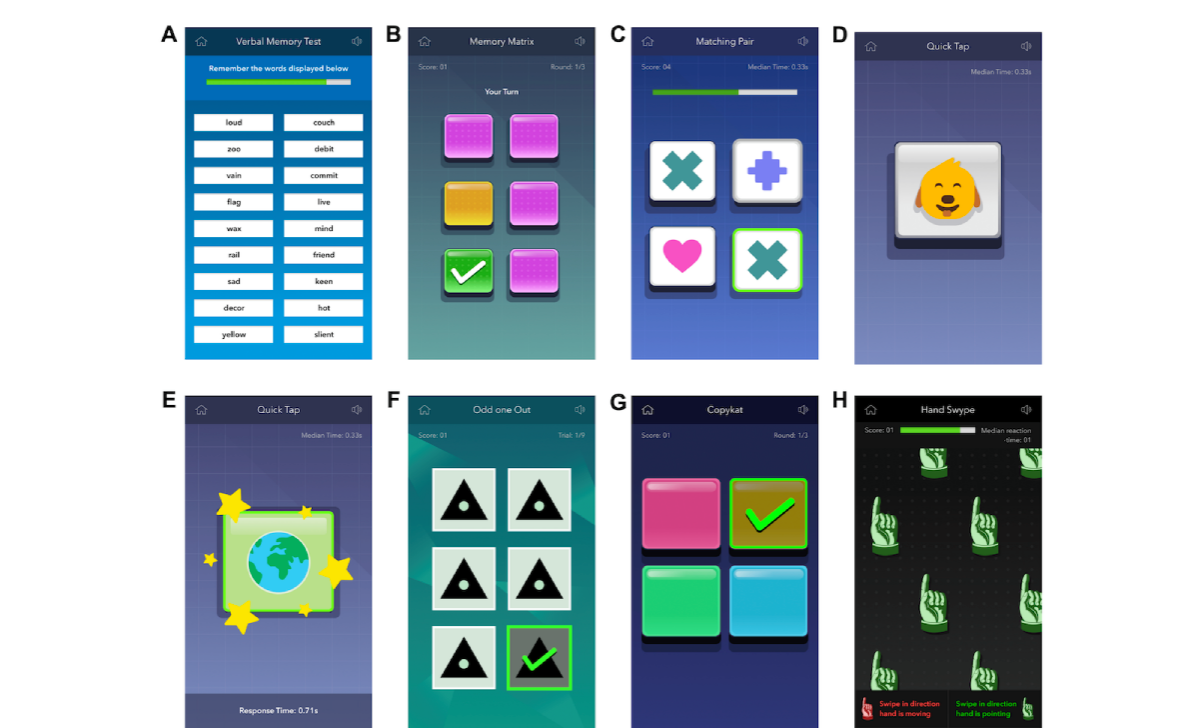
Screenshots of each NeuroUX mobile cognitive test. (A) Memory List. (B) Memory Matrix. (C) Matching Pair. (D) Quick Tap 1. (E) Quick Tap 2. (F) Odd One Out. (G) CopyKat. (H) Hand Swype.

This test was designed to assess recognition memory (Figure 1A). Participants are asked to learn a list of 12 words presented simultaneously for 30 seconds, after which the list of words is removed, and participants complete another NeuroUX cognitive test as a distractor task. Then, participants complete the recognition memory component of the Memory List test, which presents 24 words 1 by 1 (ie, the 12 target words mixed in with 12 distractor words). For each word, participants are asked to select “Yes” if the word was on the original list or select “No” if the word was not on the original list. The recognition trial takes approximately 1 minute to complete. Five different word lists were presented across sessions over the study period. The development of the word lists has been described previously [17]. The Memory List score is the total number of correct hits of target words and correct rejections of distractor words, with a total possible score of 24. Memory List scores of 0 were excluded due to suspected low effort or engagement.

#### Memory Matrix

This test was designed to assess visual working memory (Figure 1B). Participants are shown a grid of tiles, with the number of tiles increasing gradually across correct trials. For each grid shown, some tiles are highlighted simultaneously for 1.5 seconds. When the highlighted color returns to the original tile color, participants are asked to select all tiles that were highlighted. The number of highlighted tiles increases with grid size across correct trials. Memory Matrix takes 1 to 2 minutes to complete. The total score represents the total number of tiles selected across all correct trials. The score does not change on trials when an incorrect response is given. Memory Matrix scores were excluded for instances in which participants were not able to move beyond >3 highlighted tiles, suggesting low effort or engagement.

#### Matching Pair

This test was designed to assess processing speed (Figure 1C). Participants are shown a matrix of tiles that each display various shapes of different colors. Participants are asked to select the 2 matching tiles as quickly as possible. The matrix gradually increases in size. Scores are calculated by adding total grid sizes of correct trials. The score does not change for incorrect responses. The task ends after 90 seconds.

#### Quick Tap 1

This test was designed to assess processing speed (Figure 1D). Participants are asked to tap the target image as quickly as possible when it appears on the screen. Incorrect responses are recorded when the target image is not tapped or if the display is tapped before the target image appears. Each session contains 12 trials, with the target appearing at randomly generated time intervals of between 1 and 5 seconds since the last target was displayed. The time to complete this task is approximately 1 minute. Total correct responses are recorded, with a maximum possible score of 12. Median response times (in ms) for correct trials are also recorded as the reaction time score for each session. Different images were shown as the target image across testing sessions over the study period.

#### Quick Tap 2

This task was designed to assess response inhibition and follows a classic Go–No-Go paradigm (Figure 1E). Participants are presented with either target images that they are asked to tap as quickly as possible or foil and trick images that they are asked to refrain from tapping. Similar to Quick Tap 1, each session contains 12 trials, with the target or foil images appearing at randomly generated time intervals of between 1 and 5 seconds since the last image was displayed. The time to complete this task is 1 minute. Total correct responses are recorded, with a maximum possible score of 12, including either correctly tapping the target or not tapping the foil. Median response times (in ms) for correct target or Go trials were also recorded as the reaction time score for each session.

#### Odd One Out

This task was designed to assess both visual working memory and processing speed (Figure 1F). Participants are presented with 6 symbols, 5 of which are identical, and 1 is slightly different. Participants are asked to tap the symbol that is different as quickly as possible. Each session contains 9 trials and takes approximately 1 minute to complete. Total correct responses are recorded, with a maximum possible score of 9. Median response times (in ms) for correct responses are also recorded as the reaction time score for each session.

#### CopyKat

This task was designed to assess visual working memory (Figure 1G). The task operates similarly to the commercial game Simon such that in CopyKat, participants are shown a grid of colored tiles that light up 1 by 1 in a random order. Participants are asked to replicate the pattern by tapping the tiles in the same order that was shown. The grid size increases after each correct response. Responses are considered incorrect when the tiles are selected incorrectly or when there is no response within 20 seconds. The time to complete this task is approximately 2 to 3 minutes. The total score is the number of correct trials. CopyKat scores of <4 were excluded due to suspected low effort or engagement.

#### Hand Swype

This task was designed to assess cognitive flexibility (Figure 1H). Participants are shown several hands that are all pointing and moving across the screen in a particular direction (up, down, right, or left). The instructions change throughout the task and are always visible. Sometimes, participants are asked to swipe in the direction in which the hands are pointing regardless of the direction in which they are moving, and sometimes, they are asked to swipe in the direction in which the hands are moving. The proportion of errors made (ie, the number of errors/total trials) as well as the median response time (in ms) for correct trials are recorded for each session. The task ends after 60 seconds. Hand Swype median reaction times of <0.5 seconds were excluded due to suspected low effort or engagement.

#### Feedback Survey

At the completion of the 10 days of testing, participants were sent a feedback questionnaire that asked about feasibility, accessibility, and potential use.

### Ethical Considerations

All participants provided informed consent for this study through the Prolific platform. Participants’ data were fully anonymized during the data collection process, and researchers cannot access any identifiable participant information through Prolific. This study qualifies as exempt research under the 2018 Common Rule of the US Department of Health and Human Services and received exempt status approval from the University of California San Diego Institutional Review Board (811227). Data are securely stored on NeuroUX’s Health Insurance Portability and Accountability Act (HIPAA)–compliant Amazon Web Services servers located in the United States.

For each daily session completed, participants were compensated US $1.50. Those who completed ≥8 of the 10 daily sessions, as well as the final feedback survey, received an additional bonus of US $2.50.

### Statistical Analyses

Data from each test were aggregated across testing sessions within persons to assess both average performance and performance variability over the study period. Alternate form test-retest reliability was evaluated by calculating the intraclass correlation coefficient (ICC) for the 5 administrations of each test. ICC values for reliability were interpreted following published guidelines: poor (<0.50), moderate (0.50-0.75), good (0.76-0.90), and excellent (>0.90) [22]. To examine how the number of repeated administrations impacts the test-retest reliability of each test, we calculated cumulative ICCs, which represent the ICC values when including progressively more administrations. Practice effects were evaluated using linear mixed effects models. First, we examined linear changes in scores for each test over time (defined as study day). Person-specific random intercepts and effects of time were modeled. For scores with significant practice effects, mixed effects models with linear splines tested whether there was a changepoint at which improvements in performance leveled off [23]. Given the variable test-retest intervals across sessions, linear mixed effects models also examined test-retest interval as a predictor of score changes across sequential sessions for each test, covarying for session number and observed test scores from the prior session.

Next, associations between demographics and the average performance and within-person variability in performance for each NeuroUX test were evaluated using Pearson *r* correlation for continuous demographic variables (ie, age), Spearman correlation for ordinal demographic variables (ie, education), 2-tailed independent *t* tests for dichotomous demographic variables (ie, sex), and 1-way ANOVAs for multilevel categorical variables (ie, race and ethnicity). One-way ANOVAs were also used to examine differences in aggregate NeuroUX test performance by smartphone software type and version. Age-stratified analyses were subsequently conducted to understand whether relationships with sex, race and ethnicity, or smartphone type differed between younger (<50 y) and older (≥50 y) adults. To understand within-person fluctuations in NeuroUX test performance by contextual and environmental factors (reported for each session via EMA), separate linear mixed effects models were used for each test (outcome) and each time-varying contextual and environmental predictor: (1) being alone versus not alone, (2) being at home versus not at home, and (3) the time of day (before noon vs afternoon). Models examining the within-person relationship between being alone and test performance also covaried for the proportion of sessions during which participants reported being alone to appropriately parse apart between-person and within-person effects. Similarly, models examining the within-person relationship between being at home and test performance covaried for the proportion of sessions during which participants reported being at home, and models examining the within-person relationship between the time of day and test performance covaried for the proportion of sessions during which they completed the tests before noon. Person-specific random intercepts were included in all models.

Finally, data from the poststudy feedback survey were interpreted using descriptive statistics. We also examined the relationship between demographics and the ratings of how challenging the tests were using correlation for continuous variables and ANOVA for categorical variables. All analyses were conducted in R (version 4.3.0; R Foundation for Statistical Computing).

## Results

### Participant Characteristics

Demographic and smartphone type data for the overall sample and by age (decade bins) are presented in Table 2. Participants were aged 44.60 (SD 16.10) years on average, and 50.4% (198/393) were female. Of the 367 participants who provided responses about their race and ethnicity, 258 (70.3%) were White. Of note, the representation of people of color was lower in older age bins (eg, 40/43, 93% White in the oldest age bin, 70-79 y). All participants had at least 12 years of education, with the greatest representation among those who had a bachelor’s degree (138/373, 37%). Regarding smartphone type, a little more than half of the participants were Android users (212/393, 53.9%), and the remaining were iOS users (181/393, 46.1%).

Of the 40 unique test administrations that participants were asked to complete (8 tests, 5 times each), participants had a mean completion rate of 88% (SD 21%). Participants were most adherent to Matching Pair (mean completion rate 90%, SD 19%), followed by CopyKat (89%, SD 20%), Memory Matrix (88%, SD 20%), Odd One Out (88%, SD 20%), Quick Tap 1 (87%, SD 23%), Quick Tap 2 (87%, SD 23%), and Memory List (86%, SD 24%). This resulted in 20,662 tests taken by all participants over the study period.

**Table 2:** Participant characteristics.

		Overall sample (n=393)	Age bins (y)					
			20-29 (n=75)	30-39 (n=114)	40-49 (n=61)	50-59 (n=50)	60-69 (n=50)	70-79 (n=43)
Age (y), mean (SD)		44.60 (16.10)	24.77 (2.62)	34.54 (2.84)	44.20 (3.21)	53.70 (2.58)	65.10 (2.94)	72.28 (2.16)
Sex (male), n (%)		195 (49.6)	35 (46.7)	70 (61.4)	30 (49.2)	20 (40)	21 (42)	19 (44.2)
**Race and ethnicity^a^** **, n (%)**								
	Asian	21 (5.7)	11 (16.2)	7 (6.7)	3 (5.3)	0 (0)	0 (0)	0 (0)
	Black	44 (12)	13 (19.1)	15 (14.4)	6 (10.5)	5 (10.6)	2 (4.2)	3 (7)
	Hispanic or Latinx	27 (7.4)	10 (14.7)	12 (11.5)	2 (3.5)	2 (4.4)	1 (2.1)	0 (0)
	White	258 (70.3)	30 (44.1)	64 (61.5)	41 (71.9)	40 (85.1)	43 (89.6)	40 (93)
	Other	17 (4.6)	4 (5.9)	6 (5.8)	5 (8.8)	0 (0)	2 (4.2)	0 (0)
**Education level^b^** **, n (%)**								
	High school diploma or GED^c^	61 (16.4)	11 (16.3)	15 (14.1)	9 (14.5)	10 (20.4)	8 (16.3)	8 (18.5)
	Some college, no degree	75 (20.1)	19 (27.9)	20 (18.9)	12 (20.7)	9 (18.4)	9 (18.4)	6 (14)
	Associate degree	37 (9.9)	2 (2.9)	13 (12.3)	11 (19)	4 (8.2)	5 (10.2)	2 (4.7)
	Bachelor’s degree	138 (37)	31 (45.6)	43 (40.6)	20 (34.5)	18 (36.7)	15 (30.6)	11 (25.6)
	Master’s degree	49 (13.1)	3 (4.4)	12 (11.3)	5 (8.6)	7 (14.3)	7 (14.3)	15 (34.9)
	Professional degree beyond bachelor’s degree (eg, MD^d^, PhD^e^, DDS^f^, DVM^g^, JD^h^, and EdD^i^)	13 (3.5)	2 (2.9)	3 (2.8)	1 (1.7)	1 (2)	5 (10.2)	1 (2.3)
**Smartphone type**								
	iOS	181 (46.1)	46 (61.3)	49 (43)	30 (49.2)	20 (40)	20 (40)	16 (37.2)
	Android	212 (53.9)	29 (38.7)	65 (57)	31 (50.8)	30 (60)	30 (60)	27 (62.8)

^a^A total of 367 participants provided responses about their race and ethnicity.

^b^A total of 373 participants provided responses about their education level.

^c^GED: General Educational Development.

^d^MD: doctor of medicine.

^e^PhD: doctor of philosophy.

^f^DDS: doctor of dental surgery.

^g^DVM: doctor of veterinary medicine.

^h^JD: juris doctor.

^i^EdD: doctor of education.

### Average and Within-Person Variability in NeuroUX Performance

**Figure 2:**
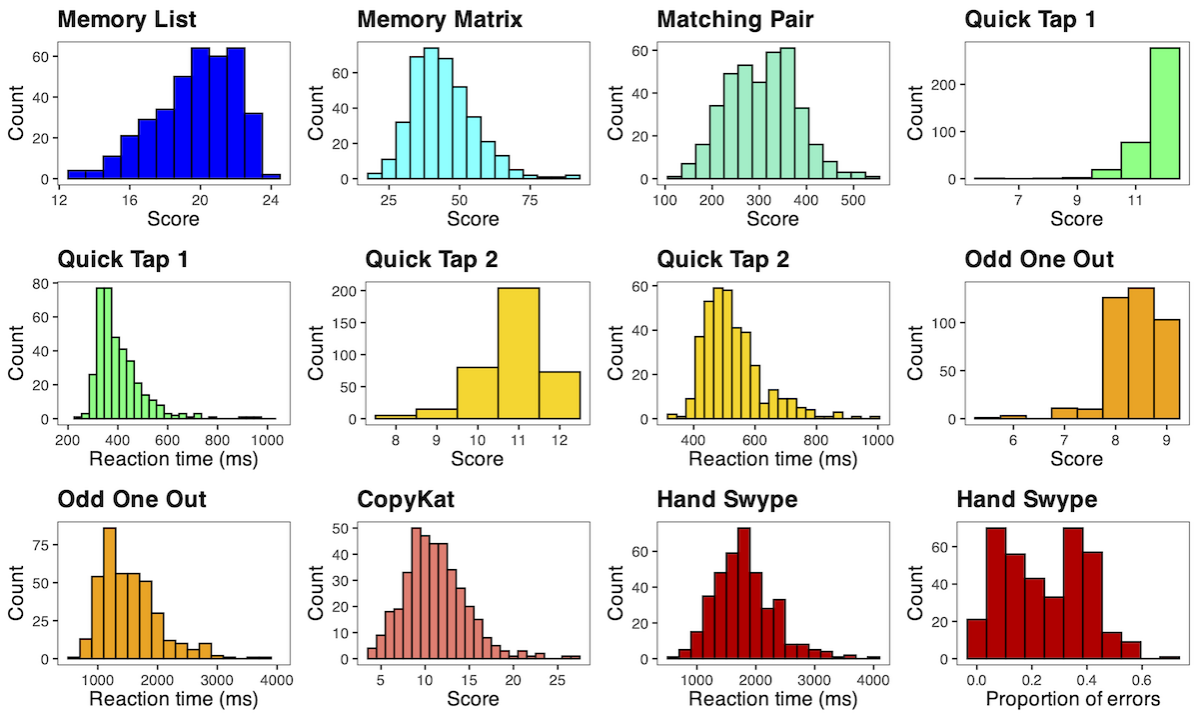
Histograms of average scores for each NeuroUX test in US adults (n=393).

Scores for each test were averaged within persons across all 5 administrations. Descriptive statistics of these average scores are reported (Table 3) to understand average performance and variability in performance between participants (histograms are presented in Figure 2). Given that participants took each test 5 times, it is also important to understand variability in tests scores within persons across administrations. SDs were calculated within persons across test administrations, and the sample average of these within-person SDs are reported in Table 4, along with the range of within-person SDs. Although the majority of within-person SDs fall below the between-person SD for each test score shown in Table 3, the maximum within-person SD values in Table 4 indicate that there were at least some participants whose performance varied widely over the study period.

**Table 3:** NeuroUX raw score distributions in US adults (n=393).

	Values, mean (SD; min-max)
Memory List score	19.8 (2.3; 12.8-24.0)
Memory Matrix score	44.3 (11.2; 21.0-89.2)
Matching Pair score	305.0 (74.5; 110.0-546.0)
Quick Tap 1 score	11.6 (0.6; 6.0-12.0)
Quick Tap 1 reaction time (ms)	404.0 (96.0; 253.8-966.5)
Quick Tap 2 score	10.9 (0.8; 7.8-12.0)
Quick Tap 2 reaction time (ms)	528.8 (98.2; 337.5-983.2)
Odd One Out score	8.4 (0.5; 5.2-9.0)
Odd One Out reaction time (ms)	1536.3 (490.5; 682.0-3784.0)
CopyKat, score	11.2 (3.5; 4.0-26.8)
Hand Swype reaction time (ms)	1808.6 (512.5; 662.4-4006.6)
Hand Swype proportion of errors	0.2 (0.1; 0.0-0.7)

**Table 4:** Within-person variability and test-retest reliability across the administrations of each NeuroUX test in US adults (n=393).

	Within-person variability				Test-retest reliability, ICC^a^
	Average within-person SD	Minimum within-person SD	Maximum within-person SD		
Memory List score	2.1	0.0	8.5	0.762	
Memory Matrix score	7.6	0.0	32.8	0.875	
Matching Pair score	52.3	7.2	134.2	0.880	
Quick Tap 1 score	0.5	0.0	4.2	0.608	
Quick Tap 1 reaction time (ms)	45.8	3.8	653.7	0.912	
Quick Tap 2 score	0.9	0.0	3.2	0.575	
Quick Tap 2 reaction time (ms)	70.5	2.9	307.3	0.865	
Odd One Out score	0.7	0.0	2.5	0.438	
Odd One Out reaction time (ms)	382.3	18.0	1550.6	0.820	
CopyKat score	3.0	0.0	9.2	0.807	
Hand Swype reaction time (ms)	339.6	15.2	1917.3	0.864	
Hand Swype proportion of errors	0.1	0.0	0.3	0.858	

^a^ICC: intraclass correlation coefficient.

### Alternate Form Test-Retest Reliability

**Figure 3:**
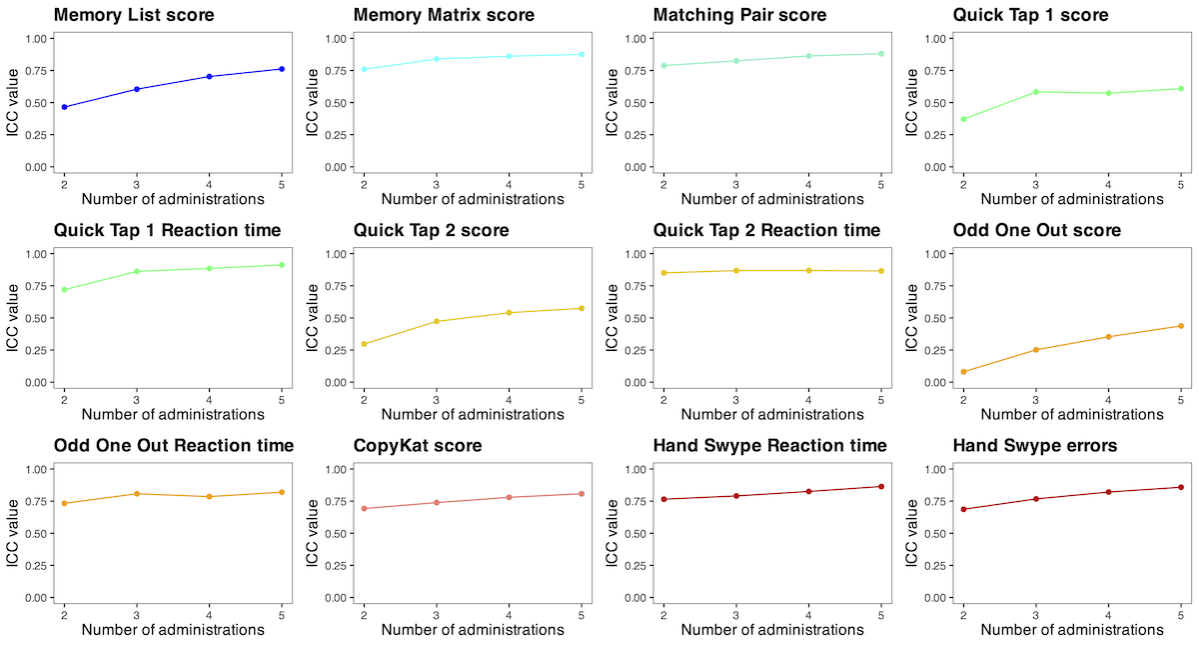
Cumulative intraclass correlation coefficients (ICCs) for each repeated NeuroUX test administration across 5 sessions in US adults (n=393).

ICCs for each test score are shown in Table 4. The majority of the test scores (9/12, 75%) showed good to excellent test-retest reliability (ICCs >0.760). However, the test scores for Odd One Out (ICC=0.438), Quick Tap 1 (ICC=0.608), and Quick Tap 2 (ICC=0.575) demonstrated poor to moderate test-retest reliability. Cumulative ICCs for each test score also showed that test-retest reliability generally increased with each repeated administration (Figure 3).

### Practice Effects

**Figure 4:**
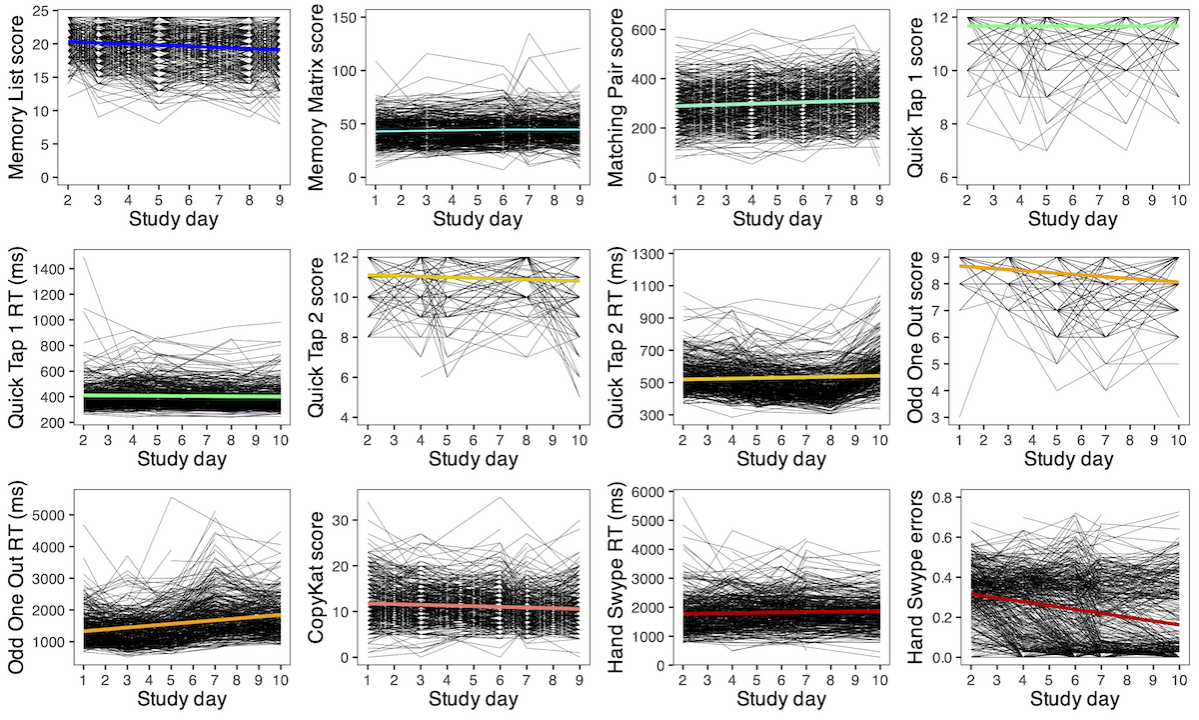
Trends in performance over time (5 sessions across 10 days) for each NeuroUX test score in US adults (n=393). RT: reaction time.

Trends over time for each test score are shown in Figure 4. Statistically significant linear practice effects (ie, improvements in performance) were observed for Memory Matrix score (*b*=0.24, SE 0.07; *P*=.001), Matching Pair score (*b*=3.69, SE 0.47; *P*<.001), Quick Tap 1 reaction time (*b*=–1.38, SE 0.53; *P*=.009), and Hand Swype proportion of errors (*b*=–0.020, SE 0.001; *P*<.001). Subsequent mixed effects regression models with linear splines showed that practice effects in Memory Matrix score leveled off after study day 6, and Quick Tap 1 reaction time leveled off after study day 8 (Multimedia Appendix 1); however, there was no point at which Matching Pair performance or Hand Swype errors significantly leveled off. Notably, several test scores (7/12, 58%) also showed that participants performed significantly worse over time on average, including Memory List score (*b*=–0.175, SE 0.020; *P*<.001), Quick Tap 2 score (*b*=–0.039, SE 0.010; *P*<.001), Quick Tap 2 reaction time (*b*=1.489, SE 0.680; *P*=.03), Odd One Out score (*b*=–0.072, SE 0.010; *P*<.001), Odd One Out reaction time (*b*=54.196, SE 3.180; *P*<.001), CopyKat score (*b*=–0.145, SE 0.030; *P*<.001), and Hand Swype reaction time (*b*=10.499, SE 3.710; *P*=.005). There was no significant change in Quick Tap 1 score over time (*b*=0.001, SE 0.006; *P*=.82). Additional analyses showed that test-retest interval was not a statistically significant predictor of change scores for any test (Multimedia Appendix 2).

### Associations With Demographics and Test-Taking Context

Older age was associated with worse average Memory Matrix score (*r*=–0.48; *P*<.001), Matching Pair score (*r*=–0.51; *P*<.001), Quick Tap 1 reaction time (*r*=0.44; *P*<.001), Quick Tap 2 reaction time (*r*=–0.48; *P*<.001), Odd One Out reaction time (*r*=0.61; *P*<.001), CopyKat score (*r*=–0.22; *P*<.001), Hand Swype reaction time (*r*=0.31; *P*<.001), and Hand Swype proportion of errors (*r*=0.12; *P*=.03). Notably, older age was also associated with better average Quick Tap 2 score (*r*=0.27; *P*<.001). Age was not significantly associated with the other average test scores (*r* values ranging from –0.019 to 0.078; *P* values >.13). We present means and SDs of average performance for each test in Table 5. Regarding within-person variability, older age was associated with greater within-person SDs for nearly all reaction time scores (3/4, 75%), including those for Quick Tap 1 (*r*=0.18; *P*<.001), Odd One Out (*r*=0.32; *P*<.001), and Hand Swype (*r*=0.14; *P*=.01). By contrast, older age was associated with lower within-person SDs (ie, less variability) on total score metrics for almost every test (6/8, 75%), including Memory List (*r*=–0.10; *P*=.04), Memory Matrix (*r*=–0.18; *P*=.001), Matching Pair (*r*=–0.29; *P*<.001), Quick Tap 1 (*r*=–0.12; *P*=.02), Quick Tap 2 (*r*=–0.19; *P*<.001), and CopyKat (*r*=–0.18; *P*<.001).

Education was significantly positively associated with average CopyKat score (ρ=0.14; *P*=.01). No other average test scores were strongly associated with education level (ρ values ranging from –0.09 to 0.09; *P* values >.08). Regarding within-person variability, higher education was associated with greater within-person SDs for Odd One Out reaction time (ρ=0.13; *P*=.02). Education was not associated with within-person variability on any other test (ρ values ranging from –0.09 to 0.07; *P* values >.08).

Sex differences in average performance were found across several tests. Women had better Memory List scores (mean 20.1, SD 2.1) than men (mean 19.4, SD 2.4; *P*=.002). Men had faster reaction times across almost all tasks compared to women, including Quick Tap 1 reaction time (mean difference 41 ms; *P*<.001), Quick Tap 2 reaction time (mean difference 36 ms; *P*<.001), and Hand Swype reaction time (mean difference 197 ms; *P*<.001). Within-person variability did not differ significantly by sex for any test score (*P* values >.06). Age-stratified analyses (<50 y and ≥50 y) showed that sex differences in average Memory List scores were driven by older adults (mean difference 1.33; *P*<.001), not younger adults (mean difference 0.44; *P*=.15), whereas sex differences in average reaction times were driven by younger adults (*P* values <.001), not older adults (*P* values >.05).

Table 6 presents differences in test performance by race and ethnicity. Differences in average scores by race and ethnicity were significantly different for Memory Matrix score, Matching Pair score, Quick Tap 1 reaction time, Quick Tap 2 score, Quick Tap 2 reaction time, Odd One Out score, Odd One Out reaction time, CopyKat score, and Hand Swype reaction time. Generally, Asian participants tended to perform best on these tasks compared to the other groups, with the exception of Odd One Out score for which the only significant pairwise difference showed that White participants scored higher than Black participants. Age-stratified analyses showed that these group differences tended to be driven by younger adults, with the exception of the significant group differences in average performance on Odd One Out reaction time, which was exhibited in both younger and older adults. Regarding within-person variability, average within-person SDs also differed by race and ethnicity for Memory List score, Memory Matrix score, Quick Tap 2 score, Odd One Out score, and Odd One Out reaction time. Group differences in within-person variability on these tasks were less consistent, with Asian participants sometimes having greater variability (ie, on Memory Matrix) and sometimes having less variability (ie, Odd One Out reaction time) than other groups. White participants also demonstrated less within-person variability than other groups on Memory List, Quick Tap 2, and Odd One Out. Again, group differences in within-person variability were all driven by younger adults; there were no significant differences in within-person variability by race and ethnicity among older adults (*P* values >.11).

Multimedia Appendix 3 presents differences in test performance by smartphone software type and version. Average scores across smartphone types were different for Memory Matrix score, Matching Pair score, Quick Tap 1 reaction time, Quick Tap 2 reaction time, Odd One Out reaction time, and CopyKat score. Participants using iOS or newer Android software versions (ie, Android 13) tended to perform better than participants with older Android software versions (eg, Android 10 or lower). These associations remained statistically significant after covarying for age, sex, education, and race and ethnicity (White vs people of color). In addition, age-stratified analyses showed consistent differences in average reaction times across smartphone type among both younger and older adults. Regarding within-person variability, average within-person SDs also differed by smartphone type for Quick Tap 1 reaction time and Odd One Out reaction time. Again, participants using newer software versions tended to be less variable than those using older versions. These associations also remained statistically significant after covarying for age, sex, education, and race and ethnicity (White vs people of color). However, in age-stratified analyses, none of the differences in within-person variability by smartphone type held in younger or older adults (*P* values >.05).

Separate linear mixed effects models for each test score showed that being at home during the NeuroUX testing session was associated with better participant performance for Matching Pair score (*b*=11.50, SE 5.18; *P*=.03), Quick Tap 1 score (*b*=0.14, SE 0.06; *P*=.02), and Quick Tap 1 reaction time (*b*=–12.46, SE 5.58; *P*=.03) compared to when participants were not at home while taking these tests. There were no significant within-person associations between being at home (vs not at home) and performance on any other tests (*P* values >.05). Similarly, linear mixed effects models showed that being alone during the NeuroUX testing session was associated with better performance for Memory List score (*b*=0.90, SE 0.20; *P*<.001), Memory Matrix score (*b*=1.92, SE 0.73; *P*=.009), and CopyKat score (*b*=0.60, SE 0.28; *P*=.03). There were no significant within-person associations between being alone (vs not alone) and performance on any other tests (*P* values >.05). Finally, linear mixed effects models showed that NeuroUX session start times before noon were associated with better performance for Odd One Out score (*b*=0.15, SE 0.05; *P*=.003) and CopyKat score (*b*=0.62, SE 0.22; *P*=.005). There were no significant within-person associations between the time of day (before noon vs afternoon) and performance on any other tests (*P* values >.05). Of note, there was a small yet significant association between older age and a higher proportion of tests taken at home (*r*=0.15; *P*=.002); however, age was not significantly associated with the proportion of tests taken alone (*r*=–0.06; *P*=.27) nor average NeuroUX session start time (ie, the hour of the day from 7 AM to 8 PM; *r*=–0.05; *P*=.36).

**Table 5:** Average NeuroUX raw scores by age.

NeuroUX test score	Age bins (y)					
	20-29 (n=75), mean (SD)	30-39 (n=114), mean (SD)	40-49 (n=61), mean (SD)	50-59 (n=50), mean (SD)	60-69 (n=50), mean (SD)	70-79 (n=43), mean (SD)
CopyKat score	11.89 (3.97)	12.00 (3.64)	10.72 (2.7)	11.72 (3.91)	10.12 (2.54)	9.26 (2.91)
Hand Swype reaction time	1641.68 (428.51)	1709.54 (410.71)	1748.54 (470.11)	1835.15 (517.44)	2085.11 (598.03)	2073.41 (611.28)
Hand Swype errors	0.24 (0.17)	0.21 (0.14)	0.28 (0.14)	0.24 (0.14)	0.26 (0.15)	0.29 (0.15)
Matching Pair score	343.58 (75.73)	336.25 (63.03)	302.27 (65.51)	284.69 (65.54)	254.30 (52.03)	237.31 (57.18)
Memory List score	19.50 (2.74)	20.06 (2.29)	19.82 (2.21)	19.69 (2.20)	19.89 (1.75)	19.41 (2.30)
Memory Matrix score	51.40 (11.40)	47.88 (10.61)	43.44 (10.57)	40.98 (9.38)	37.22 (6.85)	35.14 (6.62)
Odd One Out score	8.33 (0.56)	8.46 (0.42)	8.34 (0.58)	8.36 (0.60)	8.50 (0.35)	8.39 (0.59)
Odd One Out reaction time	1195.47 (283.89)	1344.09 (326.94)	1470.19 (346.64)	1692.96 (482.83)	1980.56 (481.33)	2038.25 (517.61)
Quick Tap 1 score	11.53 (0.53)	11.60 (0.54)	11.68 (0.39)	11.60 (0.62)	11.81 (0.28)	11.57 (1.09)
Quick Tap 1 reaction time	361.63 (59.24)	366.51 (63.28)	407.30 (80.13)	429.48 (104.47)	462.00 (119.48)	468.89 (117.34)
Quick Tap 2 score	10.46 (0.84)	10.90 (0.76)	11.01 (0.78)	11.03 (0.78)	11.19 (0.65)	11.14 (0.57)
Quick Tap 2 reaction time	484.33 (71.19)	486.62 (74.46)	524.27 (78.34)	554.99 (96.65)	615.66 (116.88)	585.35 (92.24)

**Table 6:** Aggregate performance on each NeuroUX test by race and ethnicity (n=367).

Age (y)		Asian (n=21), mean (SD)	Black (n=44), mean (SD)	Hispanic (n=27), mean (SD)	White (n=258), mean (SD)	Other (n=17), mean (SD)	*P* value	Pairwise comparisons^a^ (White >Asian, Black, Hispanic, and other)
		30.67 (7.86)	38.23 (14.49)	33.74 (10.37)	49.14 (16.04)	37.88 (12.03)	<.001	
**Average performance**								
	Memory List score	19.93 (2.39)	19.24 (2.97)	19.85 (2.35)	19.76 (2.16)	21.10 (1.73)	.09	N/A^b^
	Memory Matrix score	57.24 (18.94)	41.92 (10.27)	45.86 (9.15)	42.99 (10.25)	44.68 (8.88)	<.001	Asian >Black, Hispanic, White, and other
	Matching Pair score	349.94 (72.44)	287.76 (75.91)	322.40 (66.40)	299.41 (74.90)	335.41 (69.34)	.004	Asian >Black and White
	Quick Tap 1 score	11.65 (0.52)	11.50 (0.54)	11.68 (0.25)	11.64 (0.64)	11.58 (0.32)	.63	N/A
	Quick Tap 1 reaction time	353.88 (57.70)	428.52 (105.50)	382.18 (56.23)	410.16 (102.33)	361.89 (58.64)	.008	Asian <Black
	Quick Tap 2 score	10.64 (1.00)	10.70 (0.91)	10.79 (0.76)	11.01 (0.72)	10.72 (1.01)	.02	—^c^
	Quick Tap 2 reaction time	470.20 (38.80)	557.05 (113.38)	512.43 (75.13)	536.41 (100.12)	472.95 (82.57)	.001	Asian <Black and White
	Odd One Out score	8.39 (0.44)	8.14 (0.70)	8.42 (0.30)	8.42 (0.50)	8.46 (0.38)	.02	White >Black
	Odd One Out reaction time	1177.34 (228.32)	1755.51 (555.67)	1410.75 (327.70)	1585.60 (493.17)	1378.22 (475.68)	<.001	Asian <Black and White; Hispanic and other <Black
	CopyKat score	12.29 (4.11)	9.65 (3.55)	11.20 (2.74)	11.21 (3.60)	11.64 (2.09)	.04	Asian >Black
	Hand Swype reaction time	1564.90 (404.69)	1697.85 (504.13)	1731.54 (327.44)	1858.84 (525.08)	1818.65 (626.17)	.045	—
	Hand Swype errors	0.22 (0.16)	0.29 (0.15)	0.23 (0.13)	0.24 (0.15)	0.24 (0.15)	.32	N/A
**Within-person variability in performance (SD)**								
	Memory List score	1.97 (0.84)	2.55 (1.31)	2.66 (1.70)	2.04 (0.97)	1.61 (0.76)	.001	White and other <Black and Hispanic
	Memory Matrix score	11.88 (7.55)	7.77 (3.07)	6.66 (3.82)	7.12 (3.78)	8.40 (2.56)	<.001	Asian >Black, Hispanic, White, and other
	Matching Pair score	63.80 (25.29)	59.93 (26.02)	46.52 (22.36)	50.62 (23.24)	51.21 (17.71)	.01	—
	Quick Tap 1 score	0.56 (0.59)	0.62 (0.57)	0.43 (0.29)	0.47 (0.57)	0.47 (0.25)	.46	N/A
	Quick Tap 1 reaction time	38.51 (48.16)	45.15 (28.92)	36.62 (21.48)	48.29 (51.60)	34.61 (18.31)	.52	N/A
	Quick Tap 2 score	1.02 (0.53)	1.12 (0.70)	1.05 (0.50)	0.83 (0.50)	0.75 (0.40)	.003	White <Black
	Quick Tap 2 reaction time	67.09 (34.79)	73.83 (28.03)	72.38 (56.96)	71.11 (39.73)	58.60 (29.97)	.73	N/A
	Odd One Out score	0.71 (0.42)	0.93 (0.49)	0.77 (0.30)	0.65 (0.41)	0.67 (0.44)	.001	White <Black
	Odd One Out reaction time	252.29 (138.84)	465.28 (277.51)	342.55 (222.01)	389.48 (241.04)	358.96 (167.07)	.01	Asian <Black
	CopyKat score	3.09 (1.36)	2.98 (1.38)	2.91 (1.80)	2.92 (1.49)	3.00 (0.83)	.99	N/A
	Hand Swype reaction time	252.86 (210.82)	309.55 (223.32)	343.95 (139.10)	356.47 (277.72)	271.04 (144.03)	.26	N/A
	Hand Swype errors	0.11 (0.05)	0.11 (0.06)	0.12 (0.05)	0.11 (0.06)	0.12 (0.05)	.85	N/A

^a^Pairwise comparisons were evaluated using the Tukey honestly significant difference test for scores with omnibus group differences.

^b^N/A: not applicable.

^c^No significant pairwise differences after Tukey honestly significant difference test adjustment for multiple comparisons.

### Poststudy Feedback

Of the 393 participants, 379 (96.4%) completed the feedback survey. Feedback about NeuroUX use and experience was generally positive (Table 7). A majority of the participants agreed or strongly agreed that (1) NeuroUX is easy to use (379/379, 100%), (2) it is easy to learn to use (376/379, 99.2%), (3) they were satisfied with NeuroUX (365/379, 96.3%), (4) they enjoyed the experience (367/379, 96.8%), and (5) an app like this would be helpful to understand their thinking skills (308/379, 81.3%). Only 4.2% (16/379) of the participants endorsed privacy concerns related to NeuroUX use, and these 16 participants were evenly distributed within the age bins (ie, 2-4 participants within every age bin reported privacy concerns). In addition, 77.3% (293/379) reported being willing to share the results with their health care provider. Participants also rated how challenging they found the NeuroUX tests on a scale ranging from 1 (*not at all*) to 10 (*extremely*); the average rating was 5.7 (SD 2.1). Pearson *r* correlation showed that challenge ratings were positively associated with age (*r*=0.28; *P*<.001) such that older adults in this study found the tests more challenging. However, challenge ratings were not associated with sex (mean difference 0.07; *P*=.76), education (ρ=–0.04; *P*=.47), or race and ethnicity (all Tukey honestly significant difference–corrected pairwise comparisons; *P* values >.11). Covarying for age, challenge ratings were also associated with worse average performance on Memory List (*b*=–0.12, SE 0.06; *P*=.048) but were not significantly associated with performance on other tests.

**Table 7:** Results from the poststudy feedback survey assessing user experience with NeuroUX (n=379).

Item	Strongly disagree, n (%)	Disagree, n (%)	Neither agree nor disagree, n (%)	Agree, n (%)	Strongly agree, n (%)
Is it easy to use?	0 (0)	0 (0)	1 (0.3)	103 (27.2)	276 (72.8)
Is it easy to learn to use?	0 (0)	1 (0.3)	2 (0.5)	99 (26.1)	278 (73.4)
I am satisfied with it.	0 (0)	0 (0)	14 (3.7)	130 (34.3)	235 (62)
It was a burden to take the tests.	212 (55.9)	145 (38.3)	16 (4.2)	4 (1.1)	2 (0.5)
I enjoyed the experience.	0 (0)	1 (0.3)	12 (3.2)	147 (38.8)	219 (57.8)
An app like this would be helpful to understand my thinking skills.	2 (0.5)	9 (2.4)	60 (16)	153 (40.4)	155 (40.9)

## Discussion

### Principal Findings

This study fills a critical gap in existing literature by providing data derived from brief, repeated, and self-administered mobile cognitive tests across the adult lifespan. We presented NeuroUX data from a general sample of US adults, including average performance and within-person variability in performance for 8 tests, each with 5 in-the-wild testing sessions over 10 days. We characterized features that are particularly important for the interpretation of performance in the repeated testing format, including test-retest reliability showing good-to-excellent estimates for most of the tests (9/12, 75%) and practice effects showing variable trends in performance on each test over time. Importantly, we also characterized features that are important to consider in remote, smartphone-based testing contexts, including relationships between cognitive test performance and (1) smartphone software type showing that newer software related to better and less variable performance, as well as (2) test-taking context showing that participants performed better on some tests when they were at home, were alone, or took the tests before noon. With these data, we aim to enhance the understanding and application of mobile cognitive testing, paving the way for improved clinical decision-making, personalized interventions, and advancements in cognitive research.

These data contribute to our overarching understanding of the range of performance on this mobile testing platform among US adults and demonstrate that even a nonclinical sample of participants have a wide range of inter- and intraindividual variabilities in performance. The range of intraindividual variability, particularly given the varying contextual factors in a real-world testing format, means that it can be quite *normal* for a person’s scores during any 1 testing session to be outside of a typically expected range (eg, –1 SD to +1 SD from the mean). Furthermore, it is also necessary to consider the influence of the test administration protocol (ie, the number of times each test is administered and the time between each administration) on test performance. The vast literature on repeat neuropsychological testing suggests that practice effects are highly affected by the length of the interval between testing sessions as well as the number of exposures to testing [24-26]. Thus, it is possible that the distribution of scores presented here may be different from that in other studies using different mobile test administration schedules. While our data provide a general sense of how adults across the lifespan tend to perform on these NeuroUX tests, they may be somewhat specific to our study conditions, and researchers are encouraged to use their own control sample to make accurate comparisons against specific clinical groups. Additional studies are needed to explore how different factors related to the test administration protocol and timing may affect aggregate score estimates.

The results showed good to excellent alternate form test-retest reliability for most of the tests (9/12, 75%); however, Odd One Out score, Quick Tap 1 score, and Quick Tap 2 score reliability estimates were suboptimal. This may be related to the limited range of possible scores on these tests, as well as the tendency for participants to perform at the ceiling level. The lower estimates of alternate form test-retest reliability for these test scores negatively impact the reliability and validity of their aggregated scores. This is consistent with previous NeuroUX studies showing that these scores were not strongly related to gold standard in-person neuropsychological tests and did not discriminate clinical groups well (ie, healthy control vs bipolar disorder [19]). Notably, the reaction times for each of these tests still showed excellent alternate form test-retest reliability and showed strong associations with age and sex in expected directions [27], making it worthwhile for NeuroUX users to continue administering these tests to participants if additional reaction time measures are indicated. Furthermore, the observed increases in alternate form test-retest reliability per each additional test administration supports the repeated testing paradigm for mobile cognitive testing.

Practice effects are also very important to consider when using a repeated testing format. Although several test scores (4/12, 33%) showed expected practice effects (ie, Memory Matrix score, Matching Pair score, Quick Tap 1 reaction time, and Hand Swype proportion of errors), Quick Tap 1 score had no significant change over time, and the remaining test scores (7/12, 58%) unexpectedly showed worse performance over time. Similar decreases in performance over time have been shown previously across some NeuroUX tests [19]; however, the reason for this trend is unclear. It is possible that these decreases in performance over time may be related to unmeasured factors such as effort, fatigue, and boredom. Although it is possible that our convenience sampling approach may increase the likelihood of study disengagement over time, the consistency of these findings across other university-recruited clinical samples [19] argues against this as a primary cause. Indeed, attrition is common in longitudinal cognitive studies [28,29]. Other possibilities include factors related to the alternate forms of each test, including varying perceptions of difficulty, or possible proactive interference effects specifically on Memory List. Regardless of the underlying cause, our characterization of these trends in performance over time in a general sample of adults is a step toward improving the interpretation of NeuroUX performance in clinical populations.

The observed associations between each test and age support their validity; for example, most of the average test scores (8/12, 67%) were negatively associated with age, consistent with well-known effects of normal aging on cognitive performance [30], and support the tests’ ability to be sensitive to age-related cognitive change. Intraindividual variability in performance across administrations was also sensitive to age such that reaction time variability was positively associated with age, and total score variability was negatively associated with age. Greater intraindividual variability in reaction times with age has been consistently supported throughout the literature [31] and may even be a marker of steeper incident cognitive decline [31,32]. Although the negative association between total score variability and age was somewhat unexpected, most of the existing literature on intraindividual variability has focused on reaction time tasks or other timed measures of processing speed. While more research may be needed to understand this paradoxical relationship between the variability in speed and accuracy metrics across the lifespan, it is possible that differences in environmental contextual factors during our in-the-wild test administrations (eg, location, noise, and external distractors), differences in study engagement or conscientiousness, or selection bias by age [33] may be playing a role in these associations.

Regarding test-taking context, we found that performance was generally better when participants were at home, were alone, or took the tests before noon, although this was only statistically significant for some of the tests. Depending on specific research questions for the future use of mobile cognitive testing platforms such as NeuroUX, researchers may consider requesting that participants take tests at home and alone in the morning hours. Alternatively, researchers may want to consider incorporating even more questions into the paired EMA survey about participants’ environment while taking the tests, with the potential to covary for these contextual factors in analyses.

Participants’ feedback about their study experience was generally positive. As the use of mobile cognitive testing platforms continues to grow, understanding participant burden, willingness, difficulty, and effort to complete cognitive tests in a “burst” repeated testing schedule is crucial for interpreting their validity to accurately assess cognitive functioning. Given our use of “gamified” cognitive tests developed in conjunction with a software design team to optimize the aesthetics, presentation, and user-friendliness of NeuroUX, our platform may be particularly well suited to create a more pleasant cognitive testing experience than traditional paper-and-pencil neuropsychological testing.

### Limitations

A thorough review of study limitations is necessary to inform future research in this field. First, the convenience sampling approach to participant recruitment limits the generalizability of our sample because possible selection bias may have influenced the range of test scores captured here [34]. In addition, regarding our sample of participants, we had limited power to test differences in performance across racial and ethnic groups, particularly in the older age ranges. Furthermore, because the older age bins had very little representation from people of color, average performance from the older adults in this sample cannot be considered representative and would not reflect a culturally appropriate comparison against future older adult NeuroUX participants from minoritized populations [35]. Future directions include gathering data in more racially and ethnically diverse general adult samples.

Next, the length of our assessment burst allowed for each test to be administered only 5 times. While this may be sufficient for many future studies depending on any given research question, 5 administrations may not be sufficient to achieve the most optimal reliability and subsequent validity of the aggregated scores. In addition, this study only examined the 12-item Memory List, whereas NeuroUX also has word lists of other lengths available. In fact, the 12-item and 18-item word lists have shown similarly strong correlations with in-person, gold standard memory testing among adults with serious mental illness [17], and future studies may want to implement the 18-word list to improve sensitivity to more subtle memory difficulties. Some of the tests demonstrated ceiling effects, which limits the distribution of test scores and power to detect relationships with clinically meaningful variables (eg, cognitive impairment). On the basis of these data, those tests are being modified within the NeuroUX platform to improve their psychometric properties. Moreover, given the completely remote nature of this study and the use of a third-party recruitment source, we were limited in our ability to characterize much else about our participants beyond demographics. Future studies may benefit from collecting additional data about socioeconomic status, medical history, and perceived current functioning. Finally, as with any study using cognitive tests, we do not know whether participants were providing optimal effort on every test and every administration. Although true effort is difficult to measure, neuropsychologists in clinical practice often use performance validity tests as a proxy of effortful performance [36,37]. Additional research is needed to develop and assess potential performance validity metrics for use with mobile cognitive testing platforms.

### Conclusions

In summary, this study represents an important step forward in the field of mobile cognitive testing by characterizing performance and the psychometric properties of several NeuroUX tests in a general sample of US adults across the lifespan. Given the ability for mobile cognitive testing to be delivered completely remotely, thereby increasing access to neuropsychological services for individuals who would not otherwise have access to an in-person clinic, there is great potential for a platform such as NeuroUX to be implemented in large-scale national or international research studies. The data generated through this study may be of particular use to studies that will implement the same protocol (ie, administer 4 tests once per day for 10 days) to use as a benchmark comparison. Mobile cognitive testing may also be useful in short prospective observational studies or to provide outcome measures in clinical trials in bursts (including before treatment, during treatment, and after treatment) or administered continuously throughout a trial. In conclusion, this study’s comprehensive characterization of data on a suite of mobile cognitive tests in a general, nonclinical US adult sample highlights its potential for implementation in large-scale research studies and enabling remote access to neuropsychological services for broader populations.

## Appendix

Multimedia Appendix 1 Results of mixed effects regression models with linear splines showing practice effects leveling off for 2 NeuroUX tests: Memory Matrix score and Quick Tap 1 reaction time.

Multimedia Appendix 2 Associations between test-retest interval and change in each NeuroUX score across sequential sessions.

Multimedia Appendix 3 Aggregate performance on each NeuroUX test by smartphone software type and version.

## Abbreviations

AD: Alzheimer disease
EMA: ecological momentary assessment
HIPAA: Health Insurance Portability and Accountability Act
ICC: intraclass correlation coefficient

## Data Availability

The datasets generated and analyzed during this study are available from the corresponding author on reasonable request.
