# Supplementary material for: Characterizing Performance on a Suite of English-Language NeuroUX Mobile Cognitive Tests in a US Adult Sample: Ecological Momentary Cognitive Testing Study
Source: J Med Internet Res. 2024 Nov 25;26:e51978. doi: 10.2196/51978 (PMC11629032; doi:10.2196/51978)
Supplement: Multimedia Appendix 1 [file jmir_v26i1e51978_app1.docx]

Multimedia Appendix 1. Results of mixed effects regression models with linear splines showing practice effects leveling off for two NeuroUX tests: Memory Matrix score and Quick Tap 1 reaction time.

|  | Regression estimate (SE) | *P* value |
| --- | --- | --- |
| ***Outcome: Memory Matrix Score*** |  |  |
| Linear spline (study day 1-6) | .327 (.117) | .005 |
| Linear spline (study day 6-10) | .035 (.217) | .872 |
| ***Outcome: Quick Tap 1 RT*** |  |  |
| Linear spline (study day 1-8) | -2.277 (.756) | .003 |
| Linear spline (study day 8-10) | 1.918 (2.196) | .382 |
