# Supplementary material for: Characterizing Performance on a Suite of English-Language NeuroUX Mobile Cognitive Tests in a US Adult Sample: Ecological Momentary Cognitive Testing Study
Source: J Med Internet Res. 2024 Nov 25;26:e51978. doi: 10.2196/51978 (PMC11629032; doi:10.2196/51978)
Supplement: Multimedia Appendix 2 [file jmir_v26i1e51978_app2.docx]

Multimedia Appendix 2. Associations between test-retest interval and change in each NeuroUX score across sequential sessions

| Outcome | Unstandardized Regression Coefficient for Test-Retest Interval (SE) | *P* value |
| --- | --- | --- |
| Memory List Change Score | -.02 (.07) | .779 |
| Memory Matrix Change Score | -.36 (.33) | .283 |
| Matching Pair Change Score | 1.15 (3.76) | .759 |
| Quick Tap 1 Reaction Time Change | 1.02 (3.51) | .770 |
| Quick Tap 1 Change Score | -.02 (.02) | .217 |
| Quick Tap 2 Reaction Time Change | -.45 (2.88) | .876 |
| Quick Tap 2 Change Score | .02 (.05) | .669 |
| Odd One Out Reaction Time Change | 1.59 (16.90) | .925 |
| Odd One Out Change Score | -.03 (.03) | .351 |
| CopyKat Change Score | .16 (.11) | .146 |
| Hand Swype Reaction Time Change | -2.35 (11.79) | .842 |
| Hand Swype Proportion of Errors Change | -2.42 (3.55) | .497 |

Note. Outcomes represent the change in score or reaction time from one session to the next. Each outcome was evaluated in separate linear mixed effects models, covarying for session number and observed score from the previous session.
