# Supplementary material for: Characterizing Performance on a Suite of English-Language NeuroUX Mobile Cognitive Tests in a US Adult Sample: Ecological Momentary Cognitive Testing Study
Source: J Med Internet Res. 2024 Nov 25;26:e51978. doi: 10.2196/51978 (PMC11629032; doi:10.2196/51978)
Supplement: Multimedia Appendix 3 [file jmir_v26i1e51978_app3.docx]

Multimedia Appendix 3. Aggregate Performance on Each NeuroUX Test by Smartphone Software Type and Version. Values are mean (SD).

|  | **A.**  **Android 9 or Below** | **B.**  **Android 10** | **C.**  **Android 11** | **D.**  **Android 12** | **E.**  **Android 13** | **F.**  **iOS 15 or Below** | **G.**  **iOS 16** | **P-value** | **Pairwise** |
| --- | --- | --- | --- | --- | --- | --- | --- | --- | --- |
| n | 21 | 49 | 28 | 34 | 80 | 30 | 151 |  |  |
| Age | 50.10 (14.46) | 53.69 (17.84) | 45.11 (17.01) | 45.88 (15.55) | 42.23 (13.90) | 41.13 (13.91) | 42.53 (16.28) | <.001 | E,F,G < B |
| ***Average Performances*** |  |  |  |  |  |  |  |  |  |
| Memory List Score | 19.49 (2.37) | 19.23 (2.54) | 19.79 (2.00) | 20.27 (2.03) | 20.04 (2.34) | 20.00 (2.57) | 19.68 (2.21) | .403 |  |
| Memory Matrix Score | 40.97 (10.00) | 40.10 (9.99) | 42.38 (9.70) | 45.30 (12.97) | 46.57 (11.48) | 46.22 (13.26) | 44.54 (10.72) | .029 | E > B |
| Matching Pair Score | 272.00 (84.74) | 256.46 (56.35) | 300.14 (71.89) | 305.26 (81.19) | 317.84 (67.61) | 318.40 (88.82) | 316.73 (71.11) | <.001 | D,E,F,G > B |
| Quick Tap 1 Score | 11.69 (0.39) | 11.65 (0.97) | 11.57 (0.44) | 11.64 (0.62) | 11.64 (0.54) | 11.70 (0.44) | 11.58 (0.52) | .931 |  |
| Quick Tap 1 Reaction Time | 467.34 (156.10) | 465.09 (125.39) | 446.09 (110.63) | 430.32 (114.16) | 372.48 (60.42) | 379.87 (64.55) | 382.47 (67.01) | <.001 | E,F,G < A,B  E,G < C |
| Quick Tap 2 Score | 10.88 (0.75) | 11.04 (0.72) | 10.81 (0.69) | 10.89 (0.82) | 10.89 (0.82) | 11.16 (0.66) | 10.87 (0.82) | .542 |  |
| Quick Tap 2 Reaction Time | 586.85 (128.61) | 591.12 (115.39) | 572.72 (105.63) | 541.10 (103.14) | 495.80 (86.09) | 517.44 (83.01) | 508.77 (76.76) | <.001 | E,F,G < A,B  E,G < C |
| Odd One Out Score | 8.49 (0.47) | 8.33 (0.57) | 8.14 (0.82) | 8.47 (0.42) | 8.42 (0.45) | 8.43 (0.71) | 8.43 (0.43) | .134 |  |
| Odd One Out Reaction Time | 1683.82 (582.37) | 1815.79 (548.17) | 1602.75 (538.17) | 1654.67 (520.07) | 1438.76 (466.08) | 1467.19 (498.05) | 1453.39 (411.37) | <.001 | E,F,G < B |
| CopyKat Score | 10.23 (3.69) | 10.05 (3.50) | 10.29 (3.69) | 11.20 (3.37) | 11.76 (2.88) | 12.74 (4.19) | 11.30 (3.57) | .012 | * |
| Hand Swype Reaction Time | 1982.43 (608.57) | 1889.20 (614.80) | 1970.43 (697.82) | 1912.94 (556.05) | 1764.47 (445.95) | 1736.70 (434.83) | 1742.02 (445.92) | .091 |  |
| Hand Swype Errors | 0.24 (0.15) | 0.29 (0.14) | 0.24 (0.15) | 0.25 (0.16) | 0.23 (0.16) | 0.22 (0.14) | 0.25 (0.15) | .316 |  |
| ***Within-Person Variability in Performances (SDs)*** |  |  |  |  |  |  |  |  |  |
| Memory List Score | 2.32 (0.97) | 1.96 (1.00) | 2.24 (1.28) | 2.08 (0.93) | 2.08 (1.30) | 1.99 (1.09) | 2.19 (1.04) | .812 |  |
| Memory Matrix Score | 7.27 (3.27) | 6.49 (3.28) | 8.18 (3.92) | 8.28 (4.20) | 7.67 (4.06) | 7.94 (5.10) | 7.53 (4.67) | .551 |  |
| Matching Pair Score | 48.49 (21.13) | 46.96 (24.57) | 55.43 (22.49) | 53.87 (24.80) | 53.70 (24.52) | 53.41 (25.74) | 52.66 (22.61) | .675 |  |
| Quick Tap 1 Score | 0.41 (0.35) | 0.46 (0.69) | 0.60 (0.40) | 0.50 (0.69) | 0.44 (0.59) | 0.42 (0.44) | 0.51 (0.48) | .833 |  |
| Quick Tap 1 Reaction Time | 48.86 (29.20) | 68.08 (97.67) | 58.40 (49.40) | 41.96 (32.56) | 34.07 (19.15) | 39.11 (35.81) | 43.88 (27.66) | .003 | G < B |
| Quick Tap 2 Score | 0.86 (0.47) | 0.86 (0.57) | 1.10 (0.61) | 0.97 (0.62) | 0.88 (0.46) | 0.69 (0.45) | 0.88 (0.53) | .151 |  |
| Quick Tap 2 Reaction Time | 66.17 (33.73) | 75.25 (43.68) | 80.89 (39.55) | 81.25 (37.94) | 68.27 (41.61) | 70.67 (50.72) | 66.04 (33.61) | .283 |  |
| Odd One Out Score | 0.72 (0.49) | 0.66 (0.43) | 0.78 (0.46) | 0.67 (0.38) | 0.70 (0.41) | 0.63 (0.37) | 0.73 (0.44) | .806 |  |
| Odd One Out Reaction Time | 407.83 (268.96) | 446.57 (280.05) | 448.55 (328.63) | 482.73 (265.72) | 328.53 (214.36) | 321.34 (196.92) | 362.11 (198.63) | .005 | E < D |
| CopyKat Score | 3.22 (1.96) | 2.84 (1.41) | 2.87 (1.41) | 2.84 (1.34) | 3.01 (1.47) | 3.22 (1.71) | 2.99 (1.49) | .902 |  |
| Hand Swype Reaction Time | 382.30 (257.36) | 423.07 (348.41) | 314.64 (209.92) | 402.52 (368.11) | 290.73 (196.54) | 309.28 (207.61) | 327.10 (218.57) | .069 |  |
| Hand Swype Errors | 0.11 (0.05) | 0.11 (0.06) | 0.11 (0.05) | 0.11 (0.05) | 0.11 (0.05) | 0.11 (0.07) | 0.11 (0.06) | .998 |  |

Note. Pairwise comparisons were evaluated using Tukey HSD for scores with omnibus group differences.

* No significant pairwise differences after Tukey HSD adjustment for multiple comparisons
